# Factors associated with the incidence of revision total knee arthroplasty in Korea between 2007 and 2012: an analysis of the National Claim Registry

**DOI:** 10.1186/s12891-015-0781-1

**Published:** 2015-10-26

**Authors:** Chang Ho Shin, Chong Bum Chang, Suk-Hyun Cho, Jin Hwa Jeong, Seung-Baik Kang

**Affiliations:** Department of Orthopaedic Surgery, SMG-SNU Boramae Medical Center, Seoul National University College of Medicine, 20, Boramae-ro 5-gil, Seoul, Dongjak-gu 156-707 Republic of Korea; Review and Assessment Committee, Health Insurance Review and Assessment Service, Seoul, Republic of Korea

**Keywords:** Age, Gender, Hospital volume, Incidence, Manufacturer volume, National Claim Registry, Revision total knee arthroplasty

## Abstract

**Background:**

The number of revision total knee arthroplasties (TKAs) in Asian countries is projected to increase with the rapid growth of primary TKA. We investigated the factors associated with the incidence of revision TKA using a nationally representative database.

**Methods:**

Data collected by the Health Insurance Review Agency of Korea, from 260,068 TKA patients between 2007 and 2012, were used to estimate the incidence rate and cumulative incidence of revision TKA according to age, gender, and hospital TKA and prosthesis manufacturer volume. Age, hospital, and manufacturer volume were categorized into three groups. The incidence rates and cumulative incidences of revision TKA were computed by combining age and gender, and by combining hospital and prosthesis manufacturer volume.

**Results:**

Incidence rates per 100,000 person-years were as follows: 1) by age: < 65 years, 447.2; 65–74 years, 363.7; ≥ 75 years, 270.9, 2) by gender: male, 537.8; female, 346.1; 3) by hospital volume (procedures/year): < 20, 536.9; 20–199, 432.3; ≥ 200, 300.1; and 4) by manufacturer volume (prostheses/year): < 1500, 772.3; 1500–3999, 453.9; ≥ 4000, 345.6. The revision TKA incidence rate in young males was significantly higher compared to that in elderly females. The difference in cumulative incidence, between hospitals with an annual volume of < 20 procedures and those with a volume of 20–199 procedures, was reduced for manufacturers with an annual volume of ≥ 4000. Similarly, the difference in cumulative incidence between manufacturers with an annual volume of <1500 prostheses and those with a volume of 1500–3999 prostheses was reduced in hospitals with an annual volume of ≥ 200.

**Conclusion:**

Revision TKA incidence varied according to age, gender, and hospital and manufacturer volume. This data could inform clinical decisions and healthcare strategies.

## Background

Total knee arthroplasty (TKA) is an efficacious and cost-effective pain-relieving procedure that improves mobility and quality of life in patients with severe knee arthritis [1–3]. TKA use has increased in numerous countries, particularly in Asia, commensurate with an aging population and continued socioeconomic development [4–7]. Changes to contemporary TKA prosthesis design and surgical techniques (including a greater number of arthroplasty-specific hospitals), together with patient indications and medical circumstances, impact upon the decision to apply TKA. In a recent study, the increased rate of TKA use in Korea was markedly higher compared to developed countries [5]; revision TKA is also projected to increase commensurate with this increase in primary TKA [8, 9].

Revision TKA represents a major challenge for healthcare providers and patients, and is associated with a substantial economic burden likely to affect national medical and insurance policies [10–12]. Many studies have reported on the epidemiology of, and factors associated with, revision TKA [4, 8–10, 13–15], primarily in the context of large single-center, or multi-center, settings [4, 13, 15]. Therefore, smaller hospitals, which account for a substantial proportion of the total number of TKA procedures performed, may be underrepresented such that drawing definitive conclusions regarding national revision TKA incidence is problematic [16–18]. Several studies conducted in Norway, Australia and Sweden have addressed this issue by using national (or in the US, statewide [Medicare]) arthroplasty databases [9, 19–22].

However, to date only one Asian study has used national databases to evaluate revision TKA use [23]. Given the increasing popularity of TKA in Asia, which accounts for > 60 % of the world’s population, and the different characteristics and medical circumstances of Asian vs. Western patients, revision TKA use in this continent warrants further study [4–7].

Several reports indicate an association between TKA outcomes and the number of procedures performed at hospitals (hospital volume) [9, 17, 19, 21, 24, 25]. However, only two Asian studies have assessed the impact of hospital stay duration, cost of hospitalization and postoperative infection status, and not specifically in the context of revision TKA [6, 26]. Furthermore, to our knowledge there has been no previous investigation of the potential relationship between the frequency with which particular manufacturers’ prostheses are used (prosthesis manufacturer volume) and revision TKA incidence.

Therefore, we investigated revision TKA incidence according to age group, gender, and hospital and prosthesis manufacturer volume, using a nationally representative Health Insurance Review Agency (HIRA) database containing reimbursement records between 2007 and 2012.

## Methods

This study used a retrospective cohort design using national data collected between 2007 and 2012 by the Health Insurance Review Agency (HIRA) of Korea, a non-profit organization supported by the Korean Ministry of Health and Welfare. All Koreans are obligated to contribute to the National Health Insurance Service, and pay hospitals or clinics 20–30 % of the total cost for medical procedures excluding cosmetic surgery or novel, unproven treatments. The remaining 70–80 % of costs are recouped by hospitals and clinics upon submission of claims to the HIRA detailing the health care services provided (including diagnoses, procedures, hospitalization period, and medical costs incurred). The HIRA reviews each claim, following which the National Health Insurance Corporation issues expenses according to the decision of the HIRA. Approximately 97 % of the Korean population is enrolled in this system; the remaining 3 % are under the Medical Aid Program, which is also supervised by the HIRA such that all information concerning medical practices can ultimately be obtained from the HIRA database. The HIRA database can be regarded as a complete enumeration; the subjects of this study were essentially all of the patients undergoing primary or revision TKA in Korea during the study period. Therefore, unlike typical studies using sampling databases, we did not need to make statistical assumptions for the study population. Similarly, other studies using the HIRA database did not perform statistical calculations for assumptions [23, 27, 28].

We identified all primary total knee replacement arthroplasties performed in Korea (denoted by HIRA codes N0712 and N2072) between January 1st, 2007 and December 31st, 2012. Patients between 45 and 90 years of age at the time of their primary TKA were included, with those also undergoing revision TKA identified by the codes N1712 and N3712, regardless of the reasons for surgery. If revision TKA codes preceded primary TKA codes, the data were excluded. A material code for the femoral component (E200-) was used to identify the prosthesis manufacturer in each case.

Data from a total of 260,068 patients who underwent primary TKA during the study period were analyzed. Their mean age was 68.8 ± 6.9 years, and there were 30,388 (11.7 %) males and 229,680 (88.3 %) females.

We calculated the incidence rate (per 100,000 primary TKA patient-years) and cumulative incidence (per 100,000 primary TKA patients) of revision TKA, with the period between primary and revision TKA representing the failure time. The incidence rates and cumulative incidences of revision TKA were computed according to age group, gender, and hospital and prosthesis manufacturer volume. The cumulative incidence of revision TKA was computed by combining age and gender and by combining hospital and prosthesis manufacturer volume. There were three age groups (<65, 65–74, and ≥ 75 years), in line with a previous study that analyzed the risk factors for TKA failure [13]. When deciding the cut-off values and numbers of groups for hospital volumes or prosthesis manufacturer volumes, we attempted to determine the number of groups and cut-off values that would allow optimal subject numbers in each group and the most noticeable difference in the revision rate among the groups based on hospital volumes or prosthesis manufacturer volumes. Based on the results, hospital volume was categorized into three groups: < 20, 20–199, and ≥ 200 primary procedures/year. These cut-offs were similar to those used in a study that evaluated the cost-effectiveness of TKA according to hospital volume [29]. Similarly, prosthesis manufacturer volume was also categorized into three groups: < 1500, 1500–3999 and ≥ 4000 prostheses/year.

## Results

**Fig. 1:**
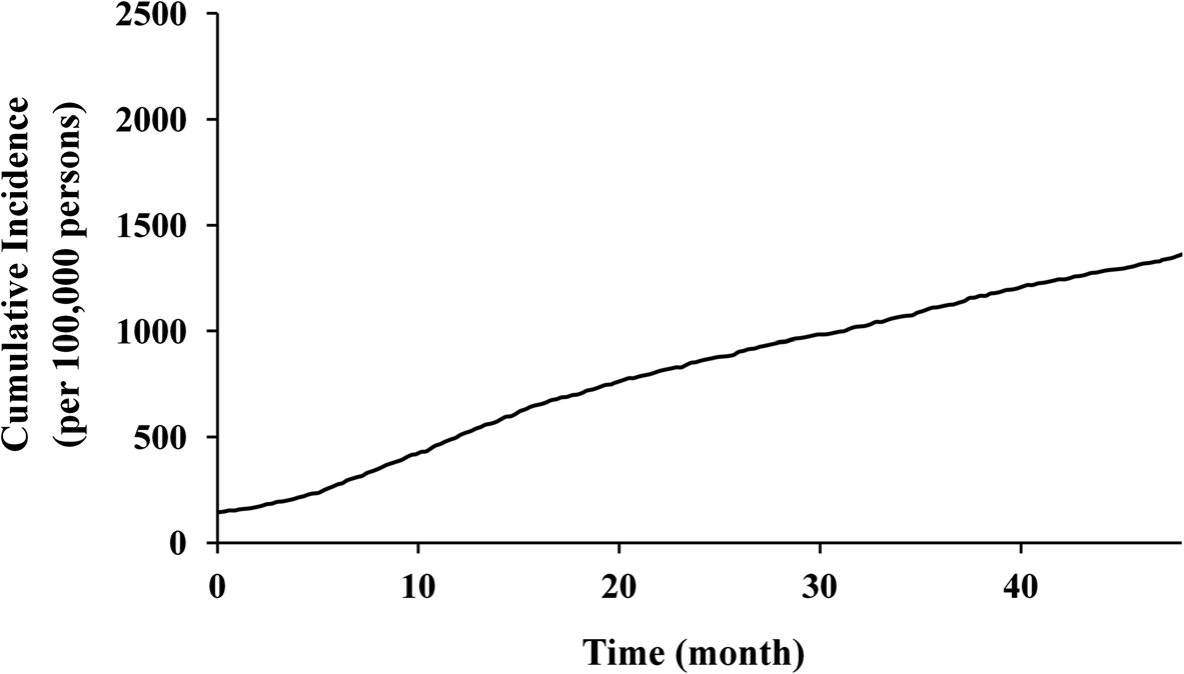
Cumulative incidence per 100,000 primary TKA patients of revision TKA procedures conducted between 2007 and 2012

During the study, 2,669 (1 %) patients received revision surgery, the cumulative incidence of which increased linearly over time (Fig. 1). Of these patients, 1,612 (60.4 %) underwent revision surgery at the same hospital at which their primary TKA was performed; in 1,257 (47.1 %) patients, the prosthesis used during their primary and revision surgeries were from the same manufacturer. In 1,045 revision surgeries, both the hospital and prosthesis manufacturer were identical to those of the primary surgery (39.2 %).

**Fig. 2:**
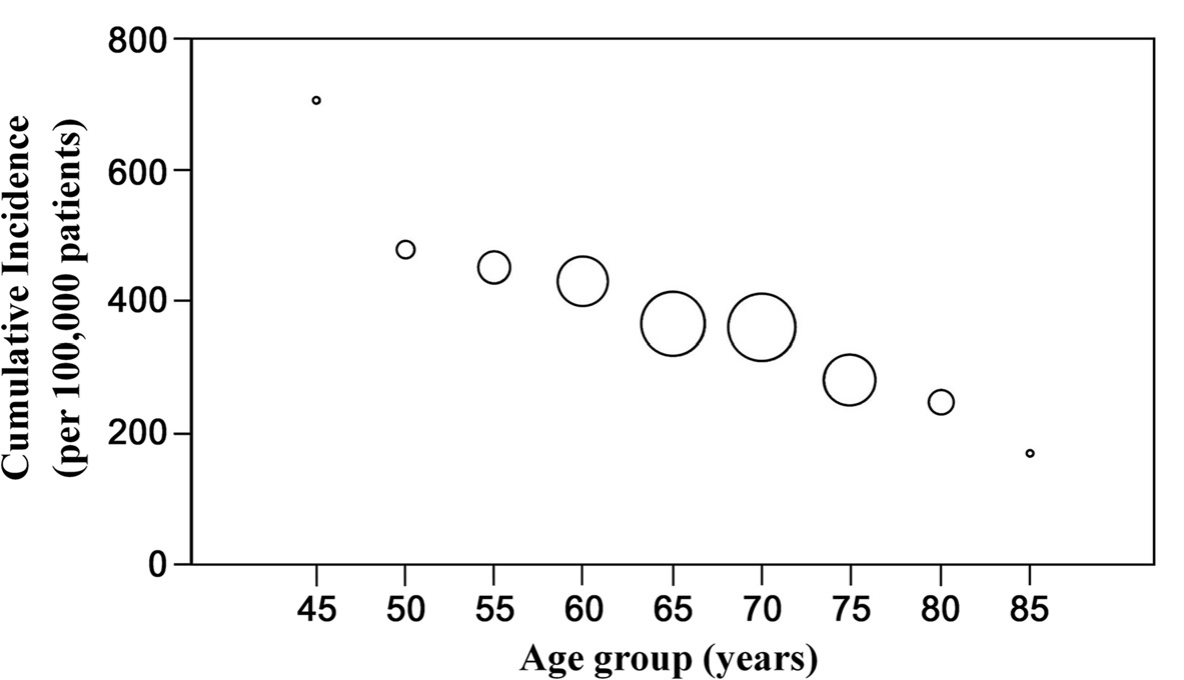
Incidence rate per 100,000 primary TKA patient-years of revision TKA by age group

**Fig. 3:**
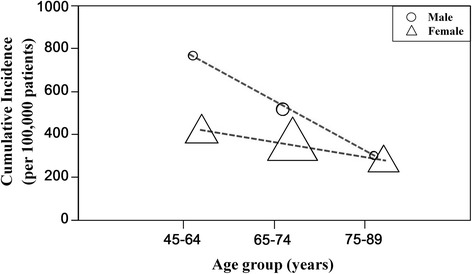
Comparison of the trends in age-specific incidence rates between males and females per 100,000 primary TKA patient-years of revision TKA

The overall incidence rate of revision TKA was 367.3/100,000 person-years; the incidence was higher in the youngest age group and males (Table 1). In the detailed analysis subdivided by age groups, the incidence in patients fifty years old or younger was extremely high (Fig. 2). When comparing the groups defined by age and gender, the youngest male group had the highest incidence rate of revision TKA (Fig. 3).

**Table 1:** Incidence of revision total knee arthroplasty according to age and gender

	Study cohort (*n* = 260,068)	
Patient factor	Number of patients (%)	Incidence rate (per 100,000 person-years)
Age		
<65 years	65,017 (25.0)	447.2
65–74 years	140,693 (54.1)	363.7
≥75 years	54,358 (20.9)	270.9
Gender		
Male	30,388 (11.7)	537.8
Female	229,680 (88.3)	346.1

**Fig. 4:**
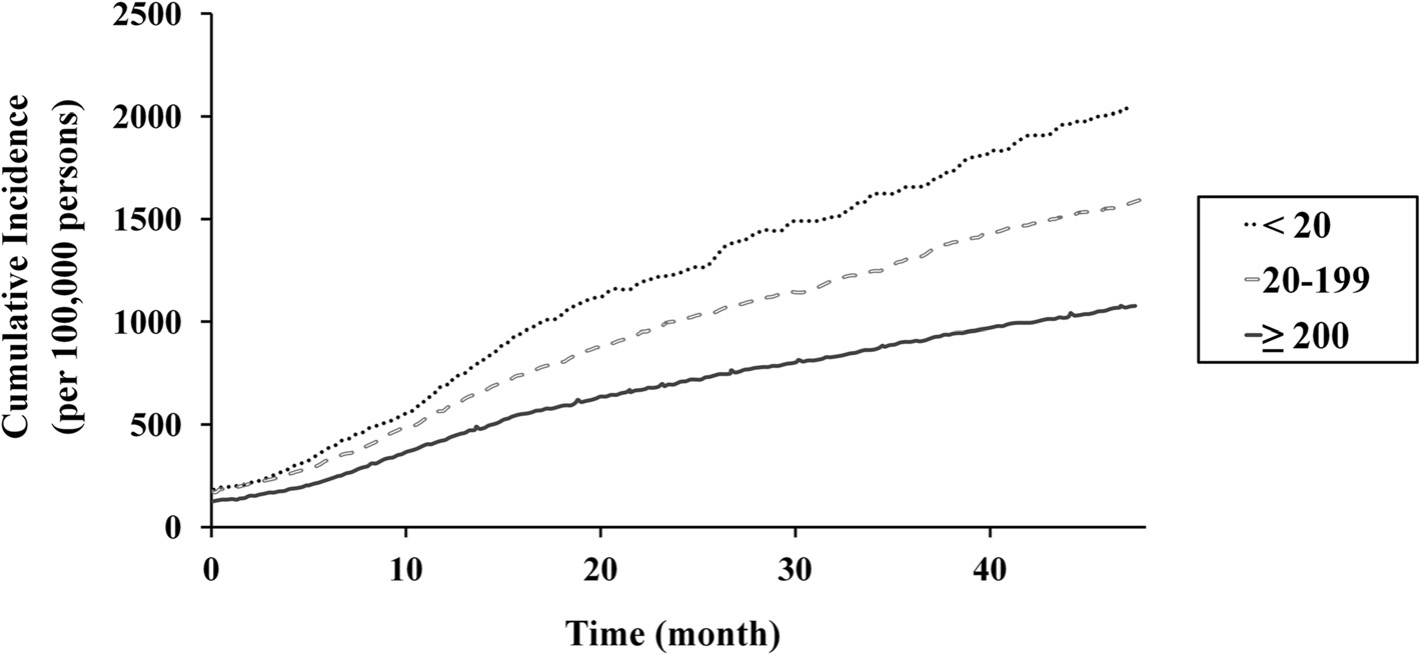
Cumulative incidence per 100,000 primary TKA patients of revision TKA in accordance with hospital volume

**Fig. 5:**
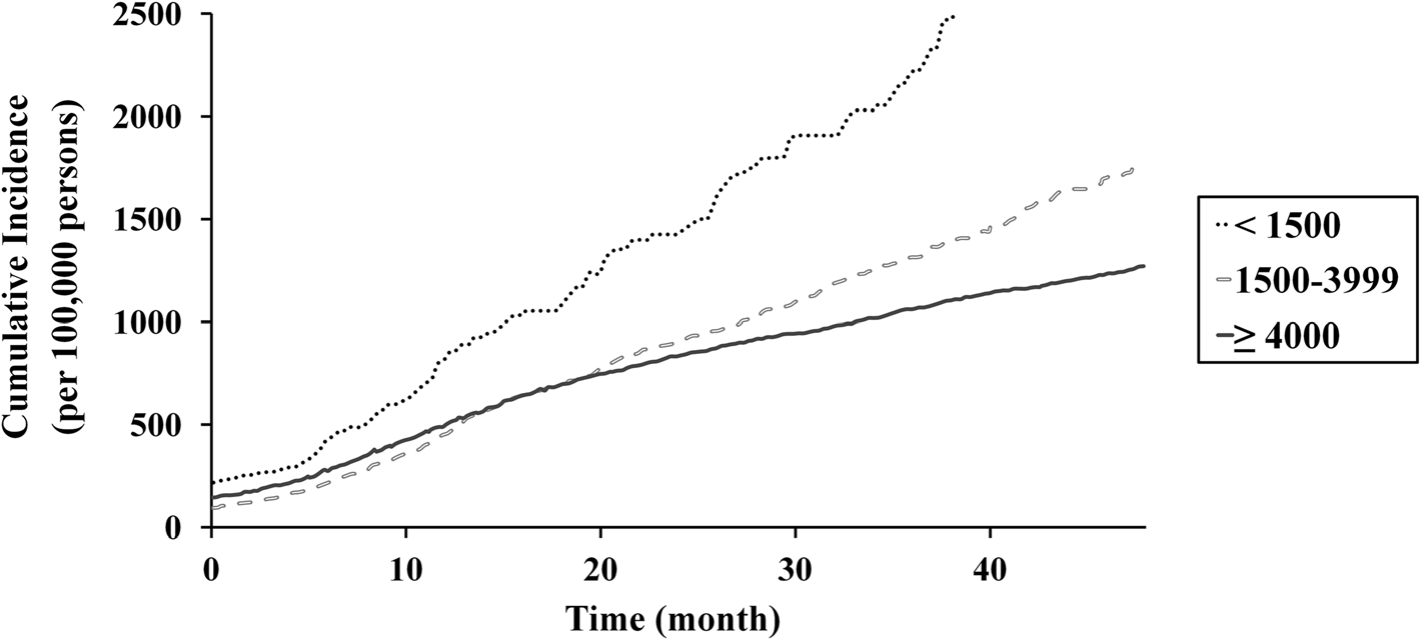
Cumulative incidence per 100,000 primary TKA patients of revision TKA in accordance with prosthesis manufacturer volume

**Fig. 6:**
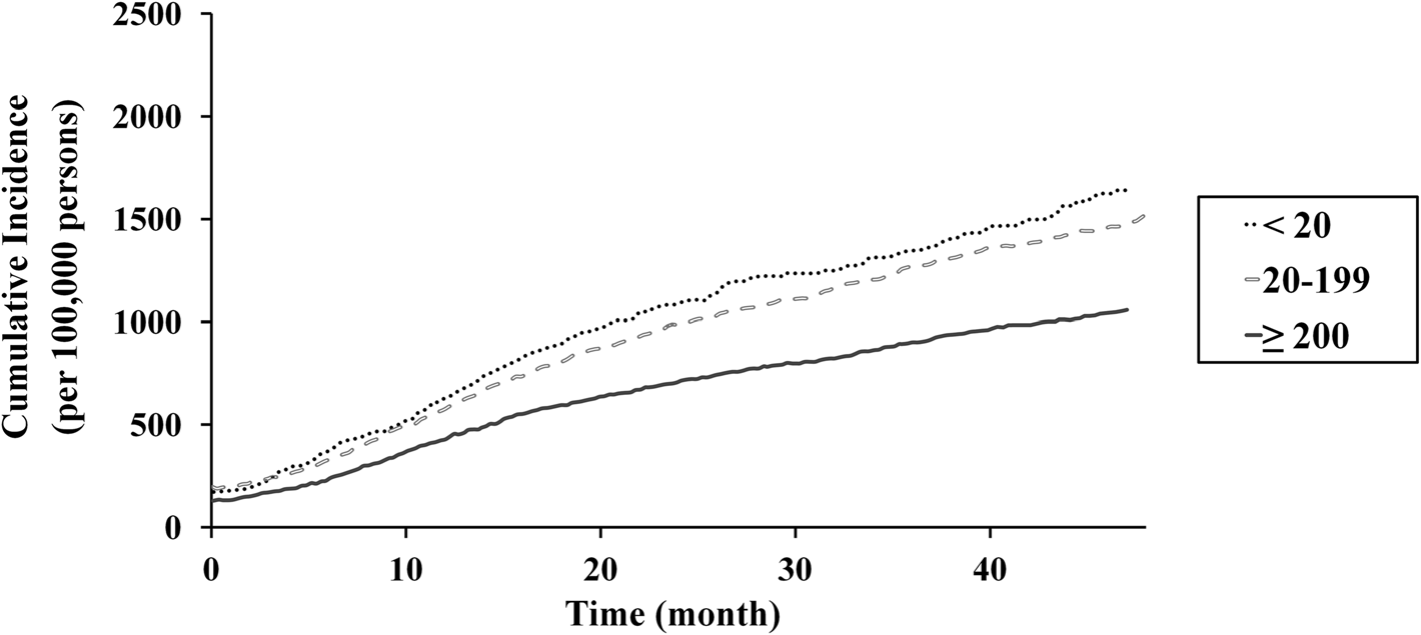
Cumulative incidence per 100,000 primary TKA patients of revision TKA in accordance with hospital volume when the prosthesis manufacturer volume was ≥ 4000 prostheses/year

**Fig. 7:**
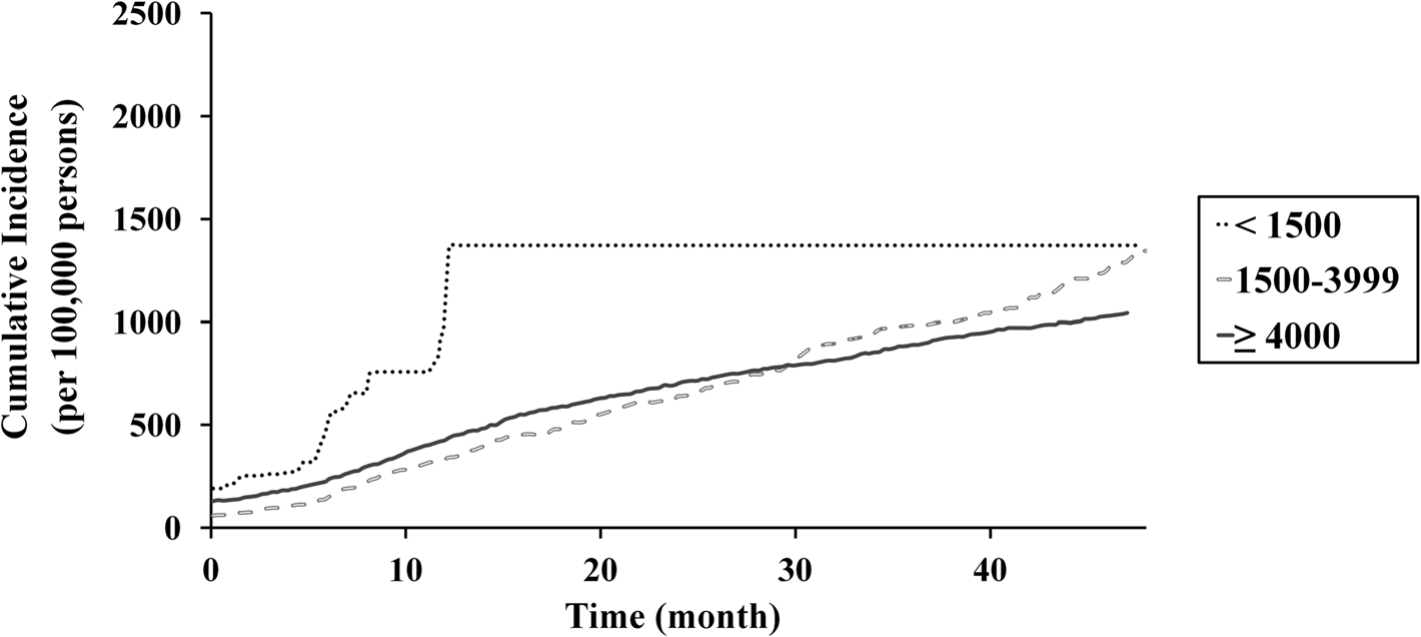
Cumulative incidence per 100,000 primary TKA patients of revision TKA in accordance with prosthesis manufacturer volume when the hospital TKA volume was ≥ 200 procedures/year

Lower hospital and prosthesis manufacturer volumes were associated with higher revision TKA incidence rates (Table 2) and cumulative incidences (Figs. 4 and 5). However, during comparison of groups defined according to hospital and prosthesis manufacturer volume, the cumulative incidence of revision TKA, of lower- (<20 procedures/year) and intermediate-volume hospitals (20–199 procedures/year) was similar when prosthesis manufacturer volume was high (≥4000 prostheses/year; Fig. 6). Similarly, the difference in cumulative incidence of revision TKA, between higher- (≥4000 prostheses/year) and intermediate- (1500–3999 prostheses/year) prosthesis manufacturer volumes, was lower in the context of higher-volume hospitals (≥200 procedures/year; Fig. 7).

**Table 2:** Incidence of revision total knee arthroplasty according to hospital and prosthesis manufacturer volume

		Study cohort (*n* = 260,068)	
Factor	Volume	Number of patients (%)	Incidence rate (per 100,000 person-years)
Hospital^a^	<20	19,335 (7.4)	536.9
	20–199	99,797 (38.4)	432.3
	≥200	140,936 (54.2)	300.1
Manufacturer^b^	<1,500	8,746 (3.4)	772.3
	1500–3999	30,153 (11.6)	453.9
	≥4000	221,169 (85.0)	345.6

^a^Mean annual number of primary total knee arthroplasties performed in each hospital during the study period

^b^Mean annual number of prostheses applied to patients undergoing primary total knee arthroplasty during the study period

## Discussion

This study evaluated information pertaining to revision TKA operations performed in Korea between 2007 and 2012, using a near-complete HIRA dataset. In particular, we assessed for the first time the association between prosthesis manufacturer volume and revision TKA incidence.

Few studies conducted in other Asian countries have dealt with the epidemiology of revision TKA. A study from Taiwan using national data reported that the number of revision TKAs increased by 23 % from 1998 to 2009 [23]. In that study, old age and male gender were associated with increases in the revision TKA rates, which concurred with our results. The proportion of females among those undergoing revision TKA was 72.2 % in the Taiwan study and 81 % in the Japan study [4], both of which were lower than our result of 88.3 %.

Higher revision rate was observed in younger patients, which accords with the results of studies of Western populations [2, 9, 15, 18, 30]. McCalden et al. [30] reported higher rates of aseptic loosening, instability, wear and/or osteolysis in younger patients, probably due to their greater levels of physical activity. Furthermore, younger patients are more likely to present with complex preoperative conditions, such as previous failed surgeries or posttraumatic deformities, which could lead to increased revision rates [30]. Surgeons may also be less likely to recommend revision TKA for elderly patients [2].

Our results also accord with those from Western countries demonstrating higher rates of revision TKA in males compared to females [2, 9, 15, 18, 31–35]; for example, Singh et al. [35] reported significantly higher rates of revision TKA, at 5 years post-primary TKA in males. Higher rates of polyethylene wear and osteolysis in male patients may be due to gender differences in knee biomechanics of and/or physical activity levels, thereby leading to higher rates of revision TKA [31, 33]. Higher infection rates in male TKA patients may also be a contributory factor [33]. Revision TKA was particularly common in younger males, such that the decision to apply TKA should be receive particularly careful consideration in this population.

Our data accord with previous studies in which lower hospital volume was associated with higher rates of revision TKA [9, 18, 19, 21, 36]. The mechanism underlying this relationship remains unknown. Kreder et al. [36] suggested that the difference in revision rates observed between lower- and higher-volume hospitals, at 1-year post-primary TKA, may be attributable to differences in expertise among healthcare providers rather than to differences in the prosthesis equipment itself. Hospital facilities and equipment, the experience of the surgeon, and the use of a selective referral system (i.e., sending patients to hospitals associated with good TKA outcomes) may all represent important factors.

Our analysis of the relationship between incidence rates and prosthesis manufacturer volume, which has not been assessed in previous studies, was similar to that observed for hospital volume. A surgeon's experience with a particular manufacturer’s prosthesis, and its design and quality, may influence revision TKA incidence. In our study, the cumulative incidences of revision TKA in intermediate- (20–199 procedures/year) and higher-volume hospitals (≥200 procedures/year) (Fig. 4) were similar to those when the prosthesis manufacturer volume exceeded 4000 prostheses/year (Fig. 6). However, we observed a decrease in the cumulative incidence of revision TKA in lower-volume hospitals (<20 procedures/year) for manufacturers with an annual volume of ≥ 4000 (Figs. 4 and 6). Combined with our other results, our findings suggest that the use of primary TKA prostheses with a higher prosthesis manufacturer volume (≥4000 prostheses/year) would reduce the revision rate, and its effect seems to be more helpful in lower-volume hospitals.

This study had several limitations. First, in cases of infection, patients managed by debridement or replacement of the polyethylene insert were not included in the study; only patients who underwent revision knee arthroplasty were included. Therefore, the TKA revision rates reported herein are not representative of the overall TKA failure rate. Second, we assessed incidence according to hospital and prosthesis manufacturer volume only, and evaluated neither the experience of surgeons nor the frequency with which a particular model of prosthesis was used. Third, the particular characteristics of Korean TKA patients (e.g., high proportion of females, small proportion of procedures performed by lower-volume hospitals [<20 procedures/year], and different living arrangements) may limit the generalizability of our data [16, 17, 37]. Fourth, although the same manufacturer produces different components, we did not distinguish the component type from a material code, but identified each manufacturer.

## Conclusions

Total knee arthroplasty performed in young, male patients, at lower-volume hospitals and with lower prosthesis manufacturer volumes, were associated with a higher incidence of revision TKA. In addition to age and gender, revision TKA incidence also varied according to hospital and prosthesis manufacturer volume. These data could inform clinical decisions and healthcare strategies; further studies are required to evaluate the association between revision TKA incidence and prosthesis manufacturer volume in other ethnic populations.

## Abbreviations

HIRA: health insurance review agency
TKA: total knee arthroplasty
